# 临床病理讨论-右肺背段空洞性病变

**DOI:** 10.3779/j.issn.1009-3419.2010.11.16

**Published:** 2010-11-20

**Authors:** 加艳 张, 殿胜 钟

**Affiliations:** 300052 天津，天津医科大学总医院呼吸科 Department of Respiratory Medicine, Tianjin Medical University General Hospital, Tianjin 300052, China

## 1 临床资料

患者，女，22岁，主因“发现右肺背段空洞3月余”于2010年5月17日收住我院。患者于入院前3个月，受凉后出现咳嗽、咳痰，黄白色粘液痰，量不多，易咳出，伴喘息，夜间为著且进行性加重，甚至不能平卧，无发热、盗汗、乏力、关节痛、肌肉痛、皮疹、胸闷、胸痛及咯血等，就诊于当地医院，胸CT示右肺下叶炎症，背段空洞性病变（图 1），肺功能为轻度阻塞性通气功能障碍，支气管舒张试验阳性，给予头孢呋辛联合左氧氟沙星治疗，症状好转出院，出院诊断为肺炎、支气管哮喘。出院后继续服用头孢丙烯及左氧氟沙星，1周后复查胸CT示右肺下叶炎症大部分消散，背段空洞依然存在（图 2）。之后患者未接受任何治疗，没有明显不适症状，能从事正常工作。

**1:**
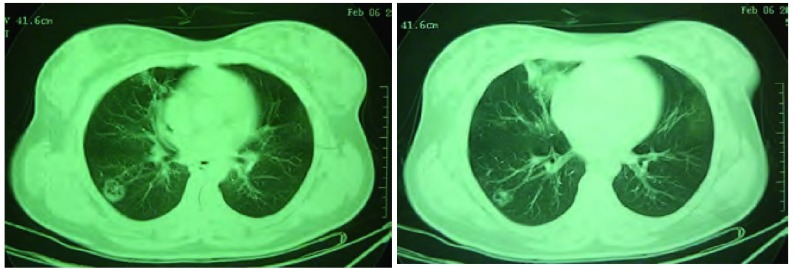
右肺下叶炎症，背段空洞性病变（2010.02.06） Inflammatory opacities in lower lobe of right lung and cavitated lesion in apical segment of lower lobe (Feb 06, 2010)

**2:**
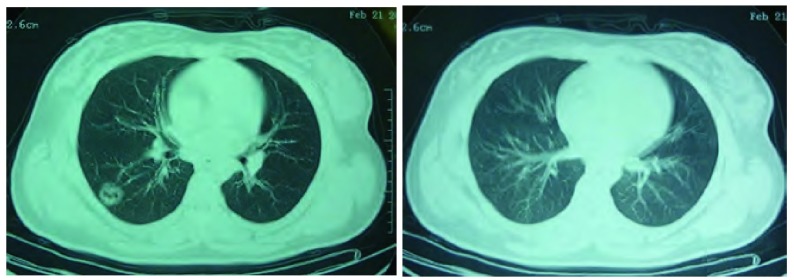
右肺下叶炎症大部分消散，背段空洞依然存在（2010.02.21） Inflammation dissipated in lower lobe of right lung after 2 week antibiotic treatment, but the cavitation was still there (Feb 21, 2010).

2010年5月17日患者就诊于我院门诊，复查胸部CT：“右肺下叶背段空洞性病变（2.3 cm×1.8 cm×2.4 cm），性质待定，不除外结核性空洞”。为求进一步诊治收入我院。患者自发病以来，精神、饮食、睡眠可，二便如常，体重、体力较前无显著变化。

既往体健，否认吸烟史，否认毒物接触及养宠物、家禽史。否认鼻炎及鼻窦炎的病史。两年前曾于服装厂工作，有棉絮、羽绒接触史。育有1子，爱人及儿子体健。无家族结核病史及遗传性疾病史。

入院查体：体温37 ℃，心率78次/分钟，呼吸17次/分钟，血压128 mmHg/70 mmHg，神清合作，全身皮肤粘膜无苍白、黄染、出血点，浅表淋巴结未触及肿大，口唇不绀，胸廓对称，双肺叩清音，双肺呼吸音清，未闻及干湿啰音，心音有力，律齐，各瓣膜区未闻及杂音，腹软，肝脾未触及，无压痛及反跳痛，双下肢不肿。

入院诊断：右肺背段空洞性病变原因待查，肺结核？肺曲霉病？

诊疗过程：入院后完善有关检查。WBC：6.9×10^9^/ L，中性粒细胞46%，淋巴细胞40.5%，RBC：4.69×10^12^/ L，HB：128 g/L，血小板：232×10^9^/L；嗜酸性粒细胞计数正常；ESR：11 mm/h；G试验：23.6 pg/mL（正常值：10 pg/mL-20 pg/mL）；肝功能、肾功能和血糖正常；痰结核菌阴性（3次）；结核抗体阴性，PPD（++）。风湿抗体（extractable nucler antigen, ENA）、抗核抗体、类风湿因子和抗中性粒细胞胞浆抗体（antineutrophil cytoplasmic antibody, ANCA）等均阴性；支气管镜检查：大致正常；支气管肺泡灌洗液检查：抗酸染色（-），真菌菌丝及孢子（-）；胸部强化CT：右肺下叶背段空洞性病变，空洞壁厚薄不均，两期强化不明显，活动性结核不除外，真菌感染待除外（图 3）。后经与患者及其家属商量，为明确诊断转入胸外科行手术探查，术中冰冻病理为腺癌（图 4A），予以右下肺楔形切除术。术后病理回报：高-中度分化腺癌，部分呈细支气管肺泡癌，部分呈微乳头腺癌，侵及胸膜（图 4B）。

**3:**
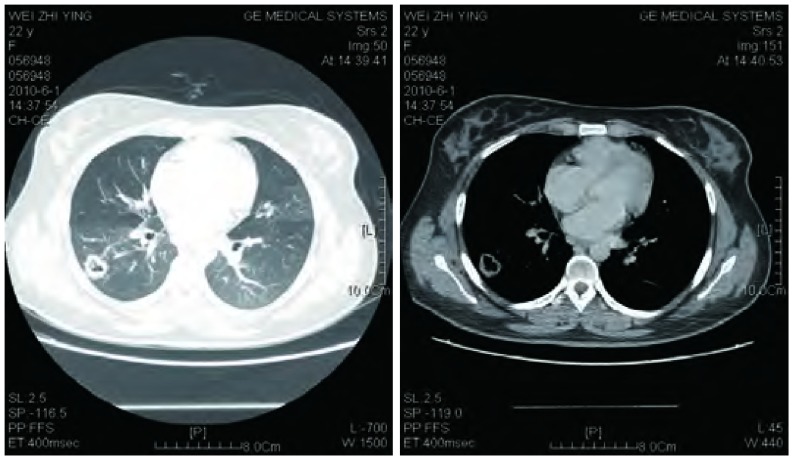
右肺下叶背段空洞性病变（2. 3 cm×1. 8 cm×2. 4 cm）（2010.06.01） The cavitation (2.3 cm×1.8 cm×2.4 cm) in apical segment of right lower lobe was no any alteration compared with 4 months ago CT finding (Jun 01, 2010)

**4:**
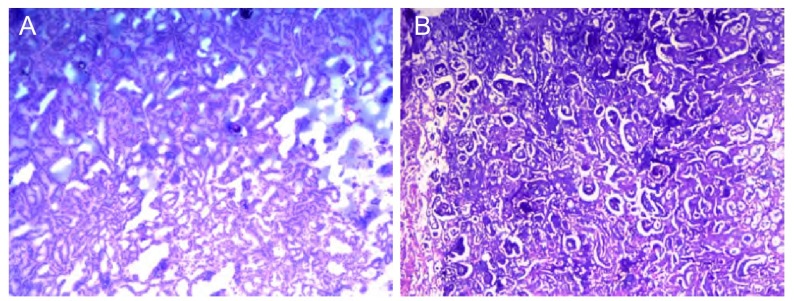
病理结果。A：术中冰冻病理为腺癌；B：术后病理为高-中度分化腺癌。 Pathological results. A: The pathological type of frozen section showed adenocarcinoma before sugical resection; B: The pathological diagnosis was well-moderately differentiated adenocardinoma in paraffin-embedded tissue.

## 2 点评

钟殿胜医师点评：该患者，年轻女性，主要临床发现为右肺下叶背段空洞3个月，我们仔细阅读了患者的胸部影像学资料，该空洞属于厚壁空洞，3个月变化不大，没有液平面和壁结节，空洞壁在强化CT扫描后无明显增强情况。导致肺部空洞常见的原因包括肿瘤性空洞（如肺癌、肺转移性肿瘤等）、感染性空洞（如结核、肺脓肿、化脓性细菌、军团菌肺炎以及真菌感染等）和肉芽肿性空洞等。首先，患者无任何关节和皮肤症状，亦无鼻部症状，尿常规、肾功能检查正常，血风湿抗体、抗核抗体和ANCA均阴性，因此，韦格纳肉芽肿和系统性红斑狼疮等胶原血管病的可能性不大。该患者以肺炎和支气管哮喘为首发表现起病，经头孢呋辛联合左氧氟沙星治疗后症状好转，未接受抗结核治疗，复查CT肺部炎症消散，但右肺背段空洞依然存在，从临床的角度考虑，患者的肺部空洞可能与此次肺部感染关系不大，基本上可以除外肺脓肿、化脓性细菌（如金黄色葡萄球菌）和军团菌肺炎等所致的肺部空洞。考虑患者年轻，空洞位于右肺下叶背段，临床上高度怀疑为结核所致的空洞，但患者3个月来无活动性结核的中毒症状，空洞无明显变化，甚至无任何明显不适和呼吸道症状，气管镜检查亦无内膜结核的表现。此外，患者出现过哮喘的症状，所以临床上还需除外肺部霉菌感染的可能，该患者没有先天或获得性免疫缺陷的病史，缺乏真菌感染的危险因素，但临床上真菌感染导致的肺部病变、甚至空洞在有些情况下比较隐袭。为进一步明确肺部空洞的原因，拟考虑做经皮肺活检或外科肺活检，经与患者及其家属商量，愿行外科手术探查。病理证实为高-中度分化腺癌，以细支气管肺泡癌成分为主。结果出乎意料。

该患者22岁，属于青年人肺癌。青年人肺癌由于处于非肺癌好发年龄，误诊率极高，有报道可达70%以上。主要原因是警惕性不够高，同样的临床表现和X线征象容易考虑肺炎或肺结核等。此外，青年人空洞型肺癌相对少见。癌性空洞是肿瘤组织因缺血坏死液化经支气管排除内容物后所形成的，常见于直径>3 cm的周围型肺癌，以鳞癌居多，约占80%，也可见于腺癌、大细胞癌和小细胞癌，肺泡癌发生空洞的机会较少。在影像学上，典型的癌性空洞通常表现为厚壁、偏心，有壁结节，周边伴有毛刺、分叶征、棘状突起等。本患者的空洞无壁结节、周边光滑无毛刺征及棘状突起等表现。这一病例提醒我们呼吸科医生要提高对年轻人肺癌的诊断意识，对于临床上诊断不清的肺外周结节或空洞应积极寻求病理学证据，如纤支镜活检、经皮肺活检、胸腔镜、外科肺活检等，达到及早诊断和治疗的目的。

